# 918. Identification of novel druggable host targets for Middle East respiratory syndrome coronavirus using a quantitative phosphoproteomics approach

**DOI:** 10.1093/ofid/ofad500.963

**Published:** 2023-11-27

**Authors:** Jasper F W Chan, Bingjie Hu, Kenn K H Chik, Shuofeng Yuan, Hin Chu

**Affiliations:** The University of Hong Kong, HONG KONG, Not Applicable, Hong Kong; The University of Hong Kong, HONG KONG, Not Applicable, Hong Kong; Centre for Virology, Vaccinology and Therapeutics, Hong Kong Science and Technology Park, HONG KONG, Not Applicable, Hong Kong; The University of Hong Kong, HONG KONG, Not Applicable, Hong Kong; The University of Hong Kong, HONG KONG, Not Applicable, Hong Kong

## Abstract

**Background:**

Middle East respiratory syndrome coronavirus (MERS-CoV) causes severe respiratory disease and extrapulmonary complications with a high mortality rate. The molecular mechanisms of MERS remain incompletely understood. Phosphoproteomics identifies proteins containing a phosphate group as posttranslational modification and allows rapid analysis of entire phosphorylation-based signaling networks. It has been increasingly used to study virus-host interactions and identify potential host-targeting treatments.

**Methods:**

Label-free quantitative phosphoproteomics on MERS-CoV-infected lung epithelial Calu-3 cells was performed. A comprehensive siRNA library knockdown screen was then performed for all upregulated phosphoproteins followed by in-silico prediction with Group-based Prediction System 3.0 to identify the top-ranked kinases for further investigations. siRNA knockdown and kinase inhibitor treatment were then performed to validate the biological relevance of the identified host targets in MERS-CoV infection. Human dipeptidyl peptidase 4-knock in (hDPP4-KI) mice were used to evaluate the treatment effects of selected inhibitors.

**Results:**

We identified more than 300 phosphopeptides that showed a change in phosphorylation status. This markedly perturbed host protein phosphorylation profile included many proteins that were involved in various cellular processes. siRNA library knockdown screen of all upregulated phosphoproteins followed by *in silico* prediction identified the 5 top-ranked kinases for further investigations. Both siRNA knockdown and kinase inhibitor treatment of these kinases resulted in the significant MERS-CoV load reduction *in vitro*. Treatment of hDPP4-KI mice with selected kinase inhibitors led to improved clinical outcome with significantly higher survival rate, less body weight loss, and lower lung viral burden.

**Conclusion:**

Quantitative phosphoproteomics is a useful platform for identifying host factors involved in MERS-CoV infection. Inhibition of these host factors may be a potential treatment strategy for this highly pathogenic coronavirus infection.

**Disclosures:**

**All Authors**: No reported disclosures

